# Comparison of Serum TARC Levels at Term‐Equivalent Age Between Preterm and Term Infants

**DOI:** 10.1155/jimr/3984014

**Published:** 2026-05-29

**Authors:** Ayako Goto, Kei Kubota, Takashi Sakaguchi, Takahiko Hirai, Yuko Horikawa, Yuma Onagawa, Yugo Nagayama, Hiroyasu Kawano, Toshikazu Niimi, Takashi Setoue, Makoto Tsutsumi, Koji Ide, Eiji Ohta, Shinichiro Nagamitsu

**Affiliations:** ^1^ Department of Pediatrics, Faculty of Medicine, Fukuoka University, Fukuoka, Japan, fukuoka-u.ac.jp; ^2^ Kubota Kids Allergy Clinic, Fukuoka, Japan; ^3^ Division of Neonatology, Center for Maternal, Fetal and Neonatal Medicine, Fukuoka University Hospital, Fukuoka, Japan, fukuoka-u.ac.jp; ^4^ Kirin Clinic, Saga, Japan; ^5^ Ide Kids Allergy Clinic, Fukuoka, Japan; ^6^ Department of Pediatrics, Fukuoka Sanno Hospital, Fukuoka, Japan

**Keywords:** CCL17 protein, chemokines, gestational age, human, hypersensitivity, immune system development, longitudinal studies, premature

## Abstract

**Objective:**

Preterm infants have a lower incidence of allergies; however, data on serum levels of thymus and activation‐regulated chemokine (TARC/CCL17) are limited. We aimed to compare serum TARC levels at term‐equivalent age between preterm and term infants and examine their association with allergic outcomes.

**Methods:**

We conducted a retrospective study of 1221 infants admitted to the neonatal intensive care unit between 2012 and 2018. Serum TARC levels were measured at 37–42 weeks’ term‐equivalent age. Infants were classified as preterm (extremely [<28 weeks], very [28–31], or moderate‐to‐late [32–36]), and term (≥37). Allergic outcomes at 6 years were assessed using International Study of Asthma and Allergies in Childhood (ISAAC)‐based caregiver questionnaires. Owing to the limited number of follow‐up responses, all preterm infants were analyzed collectively and compared with term infants.

**Results:**

Median TARC levels increased with gestational age: 549, 689, 944, and 1242 pg/mL (*p* < 0.001). Serum TARC levels were not associated with small‐for‐gestational‐age (SGA) status, sex, systemic corticosteroid use, antibiotic use, or emollient/topical therapy; did not differ significantly between inflammatory and noninflammatory neonates; and showed a weak positive correlation with eosinophil counts. At 6 years, preterm infants had lower atopic dermatitis (AD; *p* < 0.001) and higher bronchial asthma (BA; *p* = 0.003) prevalence, with no significant association between neonatal serum TARC levels and later allergic outcomes.

**Conclusions:**

Preterm infants had lower serum TARC levels at term‐equivalent age, showing a gestational age‐dependent immune maturation gradient. No association was observed with later allergic outcomes. Their BA may include nonatopic wheezing phenotypes, warranting cautious interpretation.

## 1. Introduction

Thymus and activation‐regulated chemokine (TARC), also known as cysteine–cysteine chemokine ligand 17 (CCL17), is a cysteine–cysteine chemokine primarily involved in T helper type 2 (Th2) immune responses [1]. TARC is constitutively expressed by thymic dendritic cells and is transiently upregulated in peripheral immune cells during inflammatory processes. It functions as a chemoattractant for C─C chemokine receptor type 4 (CCR4)‐expressing Th2‐cells and plays a central role in the pathogenesis of allergic diseases. Since its identification, TARC has become one of the most reliable biomarkers for assessing the severity of atopic dermatitis (AD) and monitoring treatment responses [2, 3].

Recently, cord blood TARC (cTARC) has been proposed as a potential early‐life biomarker reflecting the neonatal immune status. Elevated cTARC levels at birth have been associated with increased risks of food sensitization, AD, and other allergic conditions in early childhood [4–6]. However, most studies have focused on term infants, and data on cTARC or serum TARC levels in preterm populations remain limited. Therefore, it remains unclear whether TARC contributes to the distinct allergy risk profiles in preterm infants.

Interestingly, while preterm birth is associated with an increased risk of bronchial asthma (BA), it appears to confer a lower risk of AD and food allergies [7–9]. Additionally, preterm infants have immature respiratory function and a higher likelihood of later wheezing and BA [8]. However, the reported lower prevalence of AD and food allergy (FA) remains poorly understood and may be influenced by differences in immune maturation, microbiota development, and early environmental exposures [10].

Given TARC’s central role in Th2‐mediated immunity, examining neonatal serum TARC levels across gestational age groups may yield new insights into the immunological mechanisms that influence the development of allergic diseases. Therefore, the primary aim of this study was to compare serum TARC levels at term‐equivalent age between preterm and term infants. In addition, we conducted an exploratory retrospective follow‐up survey to investigate the relationship between serum TARC levels and allergic outcomes in early childhood.

## 2. Materials and Methods

### 2.1. Study Design

We conducted a retrospective cohort study of neonates admitted to the NICU at Fukuoka University Hospital between September 2012 and January 2018.

### 2.2. Study Participants

Infants with available serum TARC measurements obtained at 37–42 weeks term‐equivalent age were included. Exclusion criteria were congenital skin disorders, history of gastrointestinal surgery, chromosomal abnormalities, and non‐IgE‐mediated gastrointestinal FA (including food protein‐induced enterocolitis syndrome [FPIES]). To minimize confounding, infants with any of these conditions were excluded from the analysis. Participants were stratified into four gestational age groups: extremely preterm (<28 weeks), very preterm (28–31 weeks), moderate‐to‐late preterm (32–36 weeks), and term (≥37 weeks). To address the potential influence of acute inflammatory conditions on serum TARC levels, term neonates were classified into inflammatory and noninflammatory groups based on their primary diagnosis. The inflammatory group included confirmed infections, meconium aspiration syndrome, and respiratory disorders that may be associated with systemic inflammatory responses or perinatal stress during the neonatal period, such as respiratory distress syndrome (RDS) and transient tachypnea of the newborn, whereas infants treated empirically with antibiotics without a confirmed infection were categorized as noninflammatory.

### 2.3. Serum TARC Levels Measurement and Clinical Data Collection

Serum TARC levels were measured at 37–42 weeks term‐equivalent age as part of routine predischarge immunological assessments, which also included IgG, IgM, IgE, and peripheral blood eosinophil counts. At the time of sampling, C‐reactive protein (CRP) levels were confirmed to be negative in all infants, indicating the absence of overt systemic inflammation at term equivalent age. These assessments were conducted to evaluate immune status at term equivalent age and to support early identification and intervention for dermatological conditions. Serum TARC levels were quantified using a chemiluminescent enzyme immunoassay (CLEIA; SRL Inc., Tokyo, Japan). Clinical and demographic data extracted from medical records included gestational age, birth weight, sex, mode of delivery, small‐for‐gestational‐age (SGA) status, systemic corticosteroid therapy (for late‐onset circulatory failure or chronic lung disease), antibiotic exposure, and skin care practices (e.g., use of moisturizers or topical ointments). Histological chorioamnionitis severity was classified into four stages (0–3) based on the Blanc classification, which evaluates placental pathology, with stage 0 indicating no inflammation and stages 1–3 indicating increased severity. For subgroup analyses of TARC levels in relation to chorioamnionitis severity, data from extremely preterm and very preterm infants with available placental findings were combined.

### 2.4. Allergy Questionnaire

A paper‐based questionnaire based on the International Study of Asthma and Allergies in Childhood (ISAAC) was mailed to caregivers (Supporting Information 1: Table S1). The questionnaire was administered in Japanese and completed by a homogeneous Japanese population. Responses were accepted via postal mail or online submission. To adjust for varying ages at follow‐up, responses were standardized to reflect allergic diseases in children up to 6 years of age. Diagnoses of BA, food allergies, allergic rhinitis (AR), and allergic conjunctivitis (AC) were based on caregiver reports, with an emphasis on physician‐diagnosed cases. BA was defined according to ISAAC‐based criteria, which focus primarily on wheezing symptoms and caregiver‐reported physician diagnoses rather than objective clinical testing. Additional items assessed parental allergy history, household smoking status, and pet ownership. Owing to the limited number of questionnaire responses within each preterm subgroup, all preterm infants were analyzed collectively and compared with the term group in the follow‐up analysis. Analyses of allergic outcomes were restricted to participants who completed the follow‐up questionnaire at 6 years of age, for whom information on allergic outcomes and family history was available.

### 2.5. Statistical Analysis

All statistical analyses were performed using R version 4.4.0 (R Foundation for Statistical Computing, Vienna, Austria; RRID:SCR_001905). Categorical variables were compared using Pearson’s χ^2^ test or Fisher’s exact test as appropriate. Continuous variables were compared using the Kruskal–Wallis test. When overall group differences were significant, pairwise comparisons were performed using Dunn’s post hoc test with Holm correction.

Serum TARC levels, which were nonnormally distributed, were compared across gestational age groups using the Kruskal–Wallis test, followed by the Dunn’s post hoc test with Holm correction. Continuous data are presented as medians with interquartile ranges (IQRs). In addition, the association between gestational age (days) and serum TARC levels was evaluated using Spearman’s rank correlation analysis. To assess associations between serum TARC levels and clinical variables, a generalized linear model (GLM) with gamma distribution and log link was employed. Independent variables included gestational age, birth weight, sex, term‐equivalent age at sampling, mode of delivery, SGA status, systemic corticosteroid use, antibiotic exposure, and moisturizer/topical treatment use. Serum TARC levels were also compared across the four chorioamnionitis severity stages using the Kruskal–Wallis test and between binary groups (e.g., presence vs. absence of chorioamnionitis) using the Wilcoxon rank‐sum test. The incidence of allergic diseases was compared between preterm and term infants using Fisher’s exact test. Exploratory pairwise comparisons of allergic outcomes among gestational age groups were also performed using Fisher’s exact test. The Mann–Whitney *U* test was used to compare serum TARC levels between infants with and without allergic conditions within each gestational age group. To estimate the association between preterm birth and allergic outcomes, odds ratios (ORs) and 95% confidence intervals (CIs) were calculated using multivariable logistic regression models adjusted for maternal allergic disease, paternal allergic disease, household smoking exposure, and household pet ownership. Associations between parental allergic disease and AD were additionally examined using similarly adjusted models. ORs for background characteristics were calculated using univariable logistic regression models. To further evaluate the biological relevance of serum TARC levels, correlations between serum TARC levels and peripheral blood eosinophil counts measured at term equivalent age were assessed using Spearman’s rank correlation coefficient. Correlation analyses were conducted for the overall cohort and stratified by gestational age groups. In a sensitivity analysis within the term group, serum TARC levels were compared between inflammatory and noninflammatory subgroups using the Wilcoxon rank‐sum test, and the effect size (*r*) was calculated.

Statistical significance was defined as a two‐sided *p* value of <0.05.

### 2.6. Ethical Considerations

This study was approved by the Bioethics Review Committee of the Faculty of Medicine at Fukuoka University (Approval Number H24‐03‐002) and conducted in accordance with the Declaration of Helsinki. Written and oral informed consent was obtained from parents for participation in the follow‐up allergy questionnaire. All data were anonymized prior to the analysis.

## 3. Results

### 3.1. Participant Characteristics

**Figure 1:**
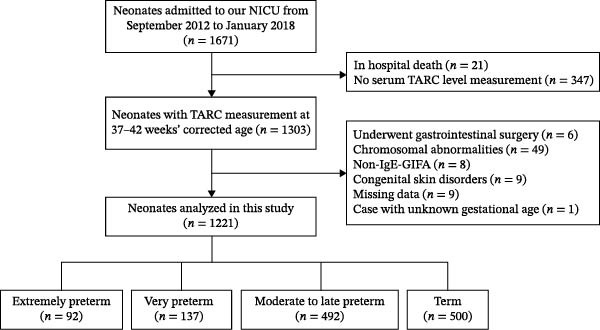
Flowchart of participant selection and classification into gestational age groups. Among 1671 neonates admitted to the NICU at Fukuoka University Hospital during the study period (September 2012–January 2018), 1221 infants with available serum TARC measurements at 37–42 weeks’ term‐equivalent age and without exclusion criteria were included in the analysis. Infants were categorized into four gestational age groups: extremely preterm (*n* = 92), very preterm (*n* = 137), moderate‐to‐late preterm (*n* = 492), and term (*n* = 500). All term infants included in this study were hospitalized neonates. GIFA, gastrointestinal food allergy; NICU, neonatal intensive care unit; TARC, thymus and activation‐regulated chemokine.

A total of 1671 neonates were initially considered for this study. Infants who died during hospitalization or who did not undergo serum TARC measurement, as well as those with congenital skin disorders, gastrointestinal surgical history, chromosomal abnormalities, non‐IgE‐mediated gastrointestinal FA (non‐IgE‐GIFA), incomplete data, or unknown gestational age, were excluded. Ultimately, 1221 neonates were included in the analysis. Among them, 92 were classified into the extremely preterm group, 137 into the very preterm group, 492 into the moderate‐to‐late preterm group, and 500 into the term group (Figure 1). In the term group, the primary reasons for hospitalization included low birth weight, respiratory disorders, jaundice, infection, and perinatal asphyxia (Supporting Information 2: Table S2). Demographic and clinical characteristics for each gestational age group are summarized in Table 1. Gestational age and birth weight differed significantly across the four groups (both *p* < 0.001), with birth weight decreasing progressively at earlier gestational ages. All pairwise comparisons for gestational age and birth weight were statistically significant. No significant differences were observed in the sex distribution (*p* = 0.205) or the proportion of SGA infants (*p* = 0.310). Vaginal delivery was significantly more frequent in the term group (60.0%) compared with preterm infants, particularly in the extremely preterm group (29.3%; *p* < 0.001). Systemic corticosteroid use was most common among extremely preterm infants (56.5%) and was not reported in moderate‐to‐late preterm or term infants (*p* < 0.001). Antibiotic exposure followed a similar pattern, being highest among extremely preterm infants (76.1%) and decreasing with advancing gestational age (*p* < 0.001). The use of moisturizers and topical ointments also decreased with gestational age, with the highest frequency observed in the extremely preterm group (83.7%; *p* < 0.001). Term equivalent age at the time of serum TARC measurement differed significantly across groups (*p* < 0.001). Post hoc Dunn tests showed significant differences between most groups, except between the extremely preterm and term groups (*p* = 0.689); the very preterm group also differed slightly from the moderate‐to‐late preterm group (*p* = 0.032). At the time of serum sampling, CRP levels were negative in all infants, indicating the absence of overt systemic inflammation at term equivalent age.

**Table 1:** Baseline characteristics by gestational age groups.

Characteristics	Extremely preterm (*n* = 92)	Very preterm (*n* = 137)	Moderate‐to‐late preterm (*n* = 492)	Term (*n* = 500)	*p* value^a^
Birth weight, median [IQR] (g)	775 (614–894)	1306 (1085–1521)	2047 (1757–2267)	2718 (2256–3095)	<0.001
Gestational age, median [IQR] (weeks)	26.1 (25–26.8)	30.3 (29.1–31.1)	34.6 (33.6–35.9)	38.4 (37.4–39.7)	<0.001
Sex: male, *n* (%)	49 (53.3%)	66 (48.2%)	286 (58.1%)	275 (55.0%)	0.205
Small for gestational age, *n* (%)	11 (12%)	25 (18.2%)	77 (15.7%)	94 (18.8%)	0.310
Delivery route: vaginal delivery, *n* (%)	27 (29.3%)	38 (27.7%)	180 (36.6%)	300 (60%)	<0.001
Systemic Corticosteroid use, *n* (%)	52 (56.5%)	8 (5.8%)	0 (0%)	0 (0%)	<0.001
Antibiotic use, *n* (%)	70 (76.1%)	53 (38.7%)	53 (10.8%)	107 (21.4%)	<0.001
Moisturizer and ointment use, *n* (%)	77 (83.7%)	76 (55.5%)	281 (57.0%)	263 (52.6%)	<0.001
Term‐equivalent age at TARC measurement, median [IQR] (weeks)	40.9 (39.4–42.9)	38.3 (37–39.9)	38 (37.1–39)	40.4 (39.4–41.7)	<0.001

Abbreviation: IQR, interquartile range.

^a^Categorical variables were compared using Pearson’s χ^2^ test or Fisher’s exact test, as appropriate, and continuous variables were compared using the Kruskal–Wallis test. Data are presented as median (IQR) or n (%). These comparisons were performed for descriptive purposes only.

### 3.2. Serum TARC Levels According to Gestational Age

**Figure 2:**
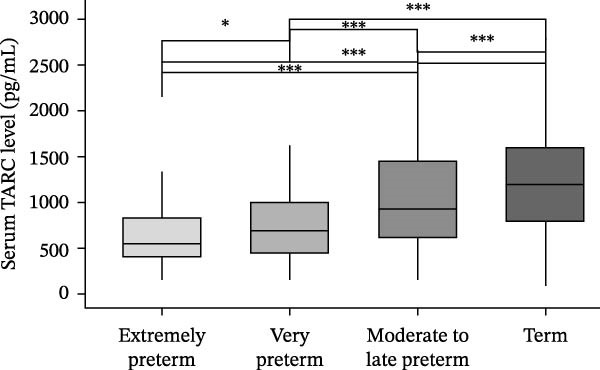
Serum TARC levels at term‐equivalent age across gestational age groups. Box plots show serum TARC levels measured at 37–42 weeks’ term‐equivalent age in four gestational age groups: extremely preterm (*n* = 92), very preterm (*n* = 137), moderate‐to‐late preterm (*n* = 492), and term (*n* = 500). Overall group differences were assessed using the Kruskal–Wallis test, followed by Dunn’s post hoc test with Holm correction for pairwise comparisons. Horizontal bars indicate statistically significant differences ( ^∗^ *p* < 0.05,  ^∗∗∗^ *p* < 0.001). The central line represents the median, box edges indicate the interquartile range (IQR), and whiskers represent 1.5 × IQR.

Serum TARC levels differed significantly across the four gestational age groups (*p* < 0.001). Median levels increased stepwise with advancing gestational age: 549 pg/mL (IQR, 402–824) in the extremely preterm group, 689 pg/mL (IQR, 449–1012) in the very preterm group, 944 pg/mL (IQR, 640–1524) in the moderate‐to‐late preterm group, and 1242 pg/mL (IQR, 833–1747) in the term group. Serum TARC levels decreased progressively with the decreasing gestational age. Post hoc Dunn’s test with Holm adjustment confirmed significant differences in all pairwise comparisons between the groups, with *p*  < 0.001 for all comparisons except between the extremely and very preterm groups (*p* = 0.019) (Figure 2). In addition, serum TARC levels showed a significant positive correlation with gestational age in days (Spearman’s *ρ* = 0.34, *p*  < 0.001). In the term group (*n* = 500), 169 neonates were classified as inflammatory and 331 as noninflammatory according to the revised definition, in which inflammatory conditions included confirmed infection and clinical conditions potentially associated with systemic inflammatory responses, such as respiratory disorders (RDS, transient tachypnea of the newborn, and meconium aspiration syndrome) and perinatal asphyxia (Supporting Information 3: Figure S3).

Serum TARC levels did not differ significantly between the inflammatory subgroup (median 1237 pg/mL, IQR 679–1766) and the noninflammatory subgroup (median 1249 pg/mL, IQR 845–1714; Wilcoxon rank‐sum test, *p* = 0.691; effect size *r* = 0.017; Supporting Information 4: Figure S4).

Eight values exceeded the 3 × IQR threshold and were classified as extreme. These extreme values were more frequently observed in the inflammatory subgroup (6/169) than in the noninflammatory subgroup (2/331; Fisher’s exact test, *p* = 0.020).

However, exclusion of these extreme values did not materially alter the comparison between inflammatory and noninflammatory neonates (Wilcoxon rank‐sum test, *p* = 0.328; effect size, *r* = 0.044).

### 3.3. Association Between Serum TARC Levels and Clinical Factors

In a GLM with a gamma distribution and log link, serum TARC levels were significantly associated with birth weight (*β* = 5.24 × 10^−4^, *p* = 0.0016), term equivalent age at sampling (*β* = −8.24 × 10^−3^, *p*  < 0.001), mode of delivery (*β* = 7.9 × 10^−5^, *p* = 0.035), and gestational age (*β* = 6.23 × 10^−3^, *p*  < 0.001). No significant associations were found between serum TARC levels and SGA status (*p* = 0.268), sex (*p* = 0.149), systemic corticosteroid use (*p* = 0.620), antibiotic exposure (*p* = 0.812), or use of moisturizers/topical treatments (*P* = 0.885; Supporting Information 5: Table S3).

### 3.4. Association Between Serum TARC Levels and Eosinophil Counts

**Figure 3:**
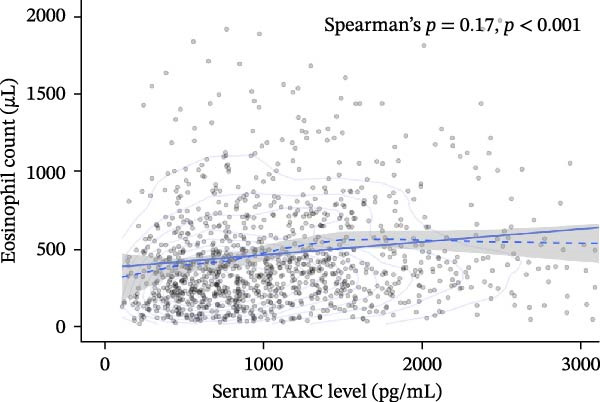
Association between serum TARC levels and peripheral blood eosinophil counts at term‐equivalent age. Scatter plot showing the relationship between serum TARC levels and peripheral blood eosinophil counts measured at 37–42 weeks of term‐equivalent age. Each point represents an individual infant. The solid line represents the fitted trend line, and the dashed line indicates locally weighted regression (LOESS). Correlation was assessed using Spearman’s rank correlation coefficient (*ρ* = 0.17, *p*  < 0.001; *n* = 1221).

Peripheral blood eosinophil counts measured at term equivalent age were analyzed to further evaluate the biological relevance of serum TARC levels. Serum TARC levels showed a significant positive correlation with eosinophil counts in the overall cohort (Spearman’s *ρ* = 0.17, *p*  < 0.001; *n* = 1221; Figure 3). When stratified by gestational age groups, the correlation was most pronounced in very preterm infants, whereas no significant correlation was observed in term infants.

### 3.5. Association Between Serum TARC Levels and Histologic Chorioamnionitis

Placental histopathological data were available for 233 infants born extremely or very preterm (Supporting Information, Section 6: Table S4). Serum TARC levels did not differ significantly according to the severity of histological chorioamnionitis (*p* = 0.467; Supporting Information 7: Figure S1). Similarly, no significant differences were observed between infants with and without histological chorioamnionitis (*p* = 0.746).

### 3.6. Follow‐Up of Allergic Development

Follow‐up questionnaires were mailed to the caregivers of 1221 children, stratified by gestational age at birth: 92 extremely preterm, 137 very preterm, 492 moderate‐to‐late preterm, and 500 term infants. Of these, 514 questionnaires were undeliverable because of relocation (21, 46, 211, and 236 patients in each group, respectively). Among the 708 questionnaires successfully sent, 257 responses were obtained (35, 39, 107, and 76 in each group, respectively), resulting in an overall response rate of 36.2%. Response rates were 49.3%, 40.7%, 37.4%, and 28.4% for the extremely preterm, very preterm, moderate‐to‐late preterm, and term groups, respectively (Supporting Information 8: Figure S2).

We compared the prevalence of allergic conditions at 6 years of age between preterm and term groups. Among children who completed the follow‐up questionnaire, the sex distribution was similar between the two groups (57.5% male in the preterm group and 57.9% male in the term group, *p* = 1.00). As shown in Table 2, the incidence of AD was significantly lower in preterm infants (*p* < 0.001), whereas ISAAC‐defined BA was significantly higher (*p* = 0.003). The prevalence of AD did not differ significantly between males and females (OR, 0.91; 95% CI, 0.38–2.24; *p* = 0.839). No significant differences were observed in the prevalence of FA, AC, or AR between the groups. Paternal allergic disease was less frequent in the preterm group than in the term group, whereas no significant differences were observed for maternal allergic disease, household smoking exposure, or household pet ownership. Adjustment for parental allergic disease and environmental factors did not materially alter the observed associations between preterm birth and allergic outcomes (Supporting Information 9: Table S5). Pairwise comparisons among gestational age groups showed similar trends, although the sample size was limited (Supporting Information 10: Table S6).

**Table 2:** Comparison of allergic diseases and related environmental and parental factors at the age of 6 years between preterm and term infants.

Characteristics	Preterm^a^ (*n* = 181)	Term (*n* = 76)	Adjusted OR (95%CI)	*p* value^b^
Male, *n* (%)	104 (57.5%)	44 (57.9%)	—	1.00
Female, *n* (%)	77 (42.5%)	32 (42.1%)	—	1.00
Food allergy, *n* (%)	15 (8.3%)	10 (13.2%)	0.60 (0.26–1.43)	0.233
Atopic dermatitis, *n* (%)	11 (6.1%)	16 (21.1%)	0.24 (0.10–0.55)	<0.001
Allergic rhinitis, *n* (%)	67 (37.0%)	35 (46.1%)	0.69 (0.40–1.19)	0.178
Bronchial asthma, *n* (%)	52 (28.7%)	8 (10.5%)	3.43 (1.62–8.17)	0.003
Allergic conjunctivitis, *n* (%)	25 (13.8%)	10 (13.2%)	1.06 (0.49–2.42)	0.889
Maternal allergic disease, *n* (%)	99 (54.7%)	50 (65.8%)	0.63 (0.36–1.09)	0.101
Paternal allergic disease, *n* (%)	81 (44.8%)	45 (59.2%)	0.56 (0.32–0.96)	0.035
Home smoker, *n* (%)	45 (24.9%)	28 (36.8%)	0.57 (0.32–1.01)	0.053
Pet ownership, *n* (%)	30 (16.6%)	14 (18.4%)	0.88 (0.44–1.81)	0.720

^a^Extremely preterm, very preterm, and moderate‐to‐late preterm groups were combined into a single preterm category due to the limited sample size in each subgroup.

^b^Fisher’s exact test was used to compare categorical variables between preterm and term infants. Adjusted for maternal allergic disease, paternal allergic disease, household smoking exposure, and pet ownership.

### 3.7. Association Between Serum TARC Levels at Term‐Equivalent Age and Allergic Diseases

We further analyzed the association between serum TARC levels at term equivalent age and the development of allergic diseases across gestational age groups. Among very preterm infants, those who developed BA had significantly higher serum TARC levels than those without asthma (*p* = 0.015). This trend was not observed in the other gestational age groups, where no significant differences in TARC levels were observed between infants with and without asthma. Similarly, no significant associations were detected between serum TARC levels and AD, FA, or AC in any gestational age group (Supporting Information 11: Table S7).

## 4. Discussion

In this study, we found that serum TARC levels measured between 37 and 42 weeks of term equivalent age were lower in infants born at earlier gestational ages. To the best of our knowledge, this is one of the first large‐scale neonatal studies to demonstrate a gestational age‐dependent gradient in serum TARC levels.

Serum TARC levels are known to be higher in neonates and infants than in adults, and several studies have reported that cTARC levels exceed postnatal levels [4, 5, 11]. Neonatal immunity is characterized by a Th2‐skewed immune profile, marked by impaired Th1 differentiation and enhanced Th2‐type responses. This Th2 bias, which reflects fetal immune tolerance to maternal antigens, is accompanied by elevated levels of Th2‐related chemokines, such as TARC (CCL17), at birth [12, 13].

Serum TARC levels showed a weak but statistically significant positive correlation with peripheral eosinophil counts at term equivalent age. This finding may support the biological relevance of TARC as a marker reflecting Th2‐related immune activity in early life. However, the strength of the correlation was modest, and the association was not consistently observed across all gestational age groups, suggesting that eosinophil counts and TARC levels may reflect only partially overlapping aspects of neonatal immune maturation. In addition, both parameters are likely influenced by gestational age and postnatal immune adaptation rather than representing a direct mechanistic link. Therefore, the observed correlation should be interpreted with caution and regarded as exploratory and hypothesis‐generating rather than as evidence of a direct causal relationship.

In the present study, serum TARC levels were measured between 37 and 42 weeks of term equivalent age, meaning that preterm infants were assessed at a later postnatal age than term infants. This difference in sampling timing may partially account for the lower serum TARC levels observed in the preterm group. However, because serum TARC levels at birth or in cord blood were not directly compared between groups, this explanation cannot be conclusively confirmed. Notably, a previous report found that cTARC concentrations in 11 infants born at 34–36 weeks ranged from 330 to 869 pg/mL, suggesting that TARC levels may already be relatively low in preterm infants at birth [5].

Additionally, multivariate analysis in our study found no significant association between serum TARC levels and treatments that could potentially influence immune responses, including systemic corticosteroids, antibiotics, or the use of moisturizers and topical agents. Therefore, it is unlikely that these interventions contributed to the observed differences in serum TARC levels among groups. Systemic corticosteroids were primarily administered during the early postnatal period, typically within the first 1–4 weeks of life (corresponding to ~25–32 weeks’ postmenstrual age) for the management of late circulatory collapse or prevention of chronic lung disease. In contrast, serum TARC levels were measured at term equivalent age (37–42 weeks’ postmenstrual age), indicating an interval of several weeks between steroid exposure and TARC assessment. Taken together, these findings suggest that a direct acute effect of systemic corticosteroids on serum TARC levels at the time of sampling is unlikely. Furthermore, all infants exhibited negative CRP levels at the time of sampling, suggesting that the observed differences in serum TARC levels were unlikely to be attributable to acute systemic inflammation.

Lower serum TARC levels observed in preterm infants may reflect immunological immaturity, particularly Th2‐cell function. Although neonatal immune responses in term infants are generally characterized by Th2 predominance, which supports tolerance induction and antibody production, previous studies suggest that preterm infants exhibit delayed Th2 development and a relatively Th1‐skewed immune profile after birth. For instance, CCR5 expression (Th1‐related) is detectable from birth, while CCR4 expression (Th2‐related) and CCR4^+^CD4^+^ cell populations increase gradually by Day 28. Furthermore, reduced IL‐4 production may downregulate Th2‐related chemokines, such as TARC, potentially explaining the lower TARC levels observed in preterm infants [14].

A study using peripheral blood mononuclear cells from women with a history of preterm delivery also showed a lower Th2/Th1 cytokine ratio, suggesting Th1 predominance in this population [15]. Similarly, preterm infants with RDS exhibit a Th1‐skewed cytokine profile, including elevated IFN‐γ and TNF‐α and reduced TGF‐β1, even after surfactant therapy, indicating persistent systemic inflammation [16]. Because TARC is a Th2‐associated chemokine, reduced levels in preterm infants may reflect this Th1‐biased immune state. However, in the present study, RDS was not evaluated, and no significant association was found between serum TARC levels and the presence or severity of histological chorioamnionitis. Therefore, we could not confirm the presence of a Th1‐skewed immune state or establish a causal relationship between this state and low TARC levels.

In the follow‐up survey of allergic diseases, the prevalence of AD was lower and that of BA was higher (*p* = 0.003) in the preterm group, consistent with previous studies [8, 9, 17]. Although preterm infants exhibited lower serum TARC levels and a lower incidence of AD, the present study did not demonstrate a significant direct association between neonatal TARC levels and subsequent AD across gestational age groups. Lower TARC levels may suggest reduced susceptibility to AD; however, this relationship should be interpreted cautiously. Despite the lower rates of atopy, preterm infants exhibited a higher prevalence of BA as defined according to ISAAC criteria. Given that asthma in preterm infants may include heterogeneous nonatopic wheezing phenotypes, this finding does not necessarily indicate a Th2‐mediated mechanism linked to TARC. Previous literature suggests that asthma susceptibility in preterm infants may involve structural lung immaturity and altered immune development rather than purely allergic mechanisms [18]. In the comparison of background characteristics, paternal allergic disease and household smoking exposure were less frequent in the preterm group than in the term group. These differences suggest potential environmental or familial confounding between the groups. However, adjustment for parental allergic disease, household smoking exposure, and pet ownership did not materially change the associations between preterm birth and allergic outcomes. The consistency between crude and adjusted estimates indicates that the observed lower incidence of AD and higher prevalence of BA in preterm infants were unlikely to be explained solely by differences in familial or environmental factors. Parental allergic disease was not independently associated with AD in adjusted analyses, further supporting that familial predisposition alone does not account for the observed differences between preterm and term infants.

Taken together, although no significant direct association was demonstrated in this cohort, lower neonatal serum TARC levels may reflect developmental differences in early Th2‐immune maturation that are relevant to atopic risk. Further prospective studies with larger sample sizes are needed to clarify the role of TARC as a marker of early immune maturation and its relationship with later allergic outcomes. Consistent with this interpretation, serum TARC levels demonstrated a significant positive correlation with gestational age in days, supporting a gestational age–dependent gradient in early immune maturation.

Consistent with these findings, exploratory analyses across gestational age subgroups demonstrated similar directional trends in allergic outcomes, with lower rates of AD and a higher prevalence of BA observed toward lower gestational ages. However, these subgroup analyses should be interpreted cautiously because of the limited sample sizes within each gestational age category.

Because all term infants in this study were hospitalized for neonatal concerns, the term group may not fully represent healthy term neonates in the general population. To address this potential limitation, we performed an additional sensitivity analysis using a revised definition of the inflammatory status. In this analysis, inflammatory conditions included confirmed infection and clinical conditions potentially associated with systemic inflammatory responses, such as respiratory disorders and perinatal asphyxia. Serum TARC levels showed nearly identical distributions between inflammatory and noninflammatory subgroups, with negligible effect size, and the exclusion of extreme values did not materially alter the results. These findings suggest that acute inflammatory conditions during the neonatal period are unlikely to have a meaningful influence on serum TARC levels in term neonates, supporting the robustness of the observed gestational age‐dependent differences.

This study has some limitations. First, the diagnoses of allergic diseases were based on caregiver‐reported questionnaires rather than physician‐confirmed evaluations, which may have introduced an information bias.

Second, BA was defined according to ISAAC‐based criteria, which primarily assess wheezing symptoms and caregiver‐reported physician diagnoses rather than objective clinical testing. In preterm infants, this definition may include heterogeneous nonatopic wheezing phenotypes related to structural lung immaturity, such as bronchopulmonary dysplasia‐associated wheeze. Therefore, respiratory morbidity in preterm infants should be interpreted cautiously and may not necessarily reflect Th2‐mediated atopic mechanisms directly linked to serum TARC levels. Therefore, the term “bronchial asthma” in this study should be interpreted as questionnaire‐defined asthma rather than clinically confirmed asthma. Third, because of the moderate response rate to the follow‐up questionnaire, allergic outcomes could not be compared across all gestational subgroups; therefore, extremely, very, and moderate‐to‐late preterm infants were combined and analyzed collectively against term infants. The relatively moderate response rate may also have introduced a response bias, and therefore, the results of the follow‐up analysis should be interpreted with caution. In addition, because the number of participants with available follow‐up data was relatively limited, particularly within each gestational age subgroup, the possibility that some observed associations may have been influenced by random variation cannot be excluded. Fourth, all term infants included in this study had been hospitalized for neonatal concerns, which may limit the generalizability of the findings to the broader healthy term population. However, sensitivity analysis within the term group demonstrated no significant difference in serum TARC levels between the inflammatory and noninflammatory subgroups. These findings suggest that acute inflammatory conditions during the neonatal period were unlikely to substantially influence serum TARC levels in term neonates. Fifth, serum TARC levels were the only Th2‐related immune marker measured, and the timing of sampling collection varied among individuals. Additionally, cTARC levels were not assessed, which precluded direct comparison between prenatal and postnatal immune profiles. Sixth, in many term infants with hospital stays shorter than 1 week, serum TARC level measurements were not performed before discharge, leading to a considerable amount of missing data. Seventh, this was a single‐center, retrospective study conducted at a university hospital, which may have limited the generalizability of the findings. In addition, clinical assessment of skin conditions at the time of blood sampling was not systematically performed for all participants, potentially influencing the interpretation of serum TARC levels, particularly in relation to the dermatological status. Furthermore, although topical therapies were not systematically used for the prevention of AD, differences in skin care practices between groups may represent a potential confounding factor in interpreting the association between serum TARC levels and AD. Eighth, residual confounding by clinical interventions could not be entirely excluded, and the observational nature of the study precluded causal inference. Finally, infants with congenital skin disorders, gastrointestinal surgery, chromosomal abnormalities, or non‐IgE‐mediated gastrointestinal food allergies were excluded. This exclusion was based on prior evidence that elevated serum TARC levels have been reported in patients with FPIES, a subtype of non‐IgE‐gastrointestinal FA [19–21]. FPIES may also occur in individuals with chromosomal abnormalities or those who have undergone gastrointestinal surgery. Furthermore, elevated TARC levels have been observed in dermatological diseases other than AD, which could confound the interpretation of TARC as a Th2‐related biomarker [22]. Accordingly, these conditions were excluded to ensure that the observed associations reflected Th2‐mediated immune activity rather than unrelated disease processes.

## 5. Conclusion

In this study, serum TARC levels were significantly lower in preterm infants than in full‐term infants, suggesting differences in early immune maturation. Although no significant association was observed between serum TARC levels and allergic diseases, preterm infants showed a lower incidence of AD. This finding is not explained by a direct association with neonatal serum TARC levels but is consistent with developmental differences in early immune maturation, reflected by lower TARC levels.

These findings highlight the potential role of TARC as a marker of early immune maturation rather than a direct predictor of allergic diseases. Further longitudinal studies incorporating cord blood analyses, comprehensive immune profiling, and allergist‐confirmed diagnostic assessments are warranted to elucidate the immunological mechanisms underlying allergic disease development in preterm infants. Although preterm infants exhibited a higher prevalence of BA, this finding should be interpreted cautiously. Because asthma in preterm infants may include heterogeneous nonatopic wheezing phenotypes related to structural lung immaturity, a direct mechanistic link between neonatal TARC levels and subsequent asthma cannot be conclusively established in this cohort.

## Funding

This study was supported by institutional funds from the Fukuoka University. No external funding was received.

## Conflicts of Interest

The authors declare no conflicts of interest.

## Supporting Information

Additional supporting information can be found online in the Supporting Information section.

## Supporting information


**Supporting Information 1** Table S1: Follow‐up questionnaire items for assessing allergic outcomes at age 6.

**Supporting Information 2** Table S2: Main diagnoses in hospitalized term infants.

**Supporting Information 3** Figure S3: Classification of term neonates into inflammatory and noninflammatory groups. Term neonates (*n* = 500) were categorized into inflammatory and noninflammatory groups based on their primary clinical diagnoses at the time of serum sampling. The inflammatory group (*n* = 169) included neonates with confirmed infection and clinical conditions potentially associated with systemic inflammatory responses, such as respiratory disorders (e.g., respiratory distress syndrome, transient tachypnea of the newborn, or meconium aspiration syndrome) and perinatal asphyxia. The noninflammatory group (*n* = 331) included neonates without these conditions.

**Supporting Information 4** Figure S4: Serum TARC levels in inflammatory and non‐inflammatory groups among term neonates. Box plots show serum TARC levels (pg/mL) in term neonates classified into inflammatory (*n* = 169) and non‐inflammatory (*n* = 331) groups based on clinical diagnoses at the time of serum sampling. Differences between groups were assessed using the Wilcoxon rank‐sum test.

**Supporting Information 5** Table S3: Generalized linear model analysis of associations between serum TARC levels and clinical variables using a gamma distribution with a log link.

**Supporting Information 6** Table S4: Severity distribution of chorioamnionitis.

**Supporting Information 7** Figure S1: Serum TARC levels according to the severity of histological chorioamnionitis. Box plots show serum TARC levels (pg/mL) across four groups based on histological chorioamnionitis severity according to the Blanc classification: stage 0 (*n* = 64), stage 1 (*n* = 56), stage 2 (*n* = 67), and stage 3 (*n* = 46). Histological chorioamnionitis data were available for 233 infants in total. Differences among groups were not statistically significant (Kruskal–Wallis test, *p* = 0.467). The central line represents the median, box edges indicate the interquartile range (IQR), and whiskers represent 1.5 × IQR.

**Supporting Information 8** SFigure S2: Questionnaire follow‐up status by gestational age groups. At 6 years of age, follow‐up questionnaires were mailed to caregivers. The numbers of questionnaires returned, undelivered because of relocation, and nonresponses are shown for each gestational age group. Response rates were 49.3% in the extremely preterm group, 40.7% in the very preterm group, 37.4% in the moderate‐to‐late preterm group, and 28.4% in the term group.

**Supporting Information 9** Table S5: Multivariable logistic regression analysis examining associations between parental allergic disease and atopic dermatitis.

**Supporting Information 10** Table S6: Prevalence of allergic diseases and environmental factors at 6 years of age by gestational age groups.

**Supporting Information 11** Table S7: Associations between serum TARC levels at term‐equivalent age and allergic outcomes at 6 years of age.

## Data Availability

The data that support the findings of this study are available upon request from the corresponding author. The data are not publicly available due to the privacy reasons.
