# Supplementary material for: Comparison of Serum TARC Levels at Term‐Equivalent Age Between Preterm and Term Infants
Source: J Immunol Res. 2026 May 29;2026:3984014. doi: 10.1155/jimr/3984014 (PMC13239061; doi:10.1155/jimr/3984014)
Supplement: Supplementary file 1 — Supporting Information 1 Table S1: Follow‐up questionnaire items for assessing allergic outcomes at age 6. [file JIMR-2026-3984014-s010.pdf]

Supplementary Table S1.
Follow-up questionnaire items for assessing allergic outcomes at age 6.

Domain	Item	Response Options
**Skin Symptoms**	Has your child ever experienced recurrent itchy rashes for more than 6 months by age 6?	Yes / No
**Skin Symptoms**	Have the itchy rashes appeared on any of the following areas: inner elbows, back of knees, front of ankles, under the buttocks, neck, ears, or around the eyes?	Yes / No
**Skin Symptoms**	At what age did the itchy rashes first appear?	Under 2 / Age 2–4 / Over 5
**Skin Symptoms**	Have the itchy rashes ever completely disappeared?	Yes / No
**Skin Symptoms**	How often did the itchy rash cause your child to wake up at night?	Never / Less than once a week / Once a week or more
**Skin Symptoms**	Has your child ever been diagnosed with atopic dermatitis by a dermatologist?	Yes / No
**Skin Symptoms**	At age 6, was your child receiving regular skin treatment?	Yes / No
**Skin Symptoms**	Is your child currently receiving skin care (moisturizing) treatment?	Yes / No
**Asthma**	Has your child ever experienced wheezing (whistling sound in the chest)?	Yes / No
**Asthma**	How many wheezing episodes has your child had per year by age 6?	None / 1–3 / 4–12 / >12
**Asthma**	How often did wheezing disturb your child's sleep by age 6?	Never / Less than once a week / Once a week or more

**Asthma**	Has your child ever experienced severe wheezing such that they could only say 1–2 words per breath by age 6?	Yes / No
**Asthma**	Has your child ever had asthma?	Yes / No
**Asthma**	Has your child ever experienced wheezing during or after exercise by age 6?	Yes / No
**Asthma**	Has your child had a dry nighttime cough unrelated to cold or pneumonia by age 6?	Yes / No
**Rhinitis**	By age 6, has your child had sneezing, runny nose, or nasal congestion unrelated to colds or flu?	Yes / No
**Rhinitis**	Did the nasal symptoms include itchy or watery eyes?	Yes / No
**Rhinitis**	In which months did the nasal symptoms occur?	Jan–Dec (check all that apply)
**Rhinitis**	How much did nasal symptoms interfere with your child's daily life?	Not at all / Slightly / Moderately / Severely
**Rhinitis**	Has your child had hay fever by age 6?	Yes / No
**Food Allergy**	At what age in months did your child begin complementary feeding?	5m–1y
**Food Allergy**	Has your child ever experienced an allergic reaction after eating specific foods by age 6?	Yes / No
**Food Allergy**	Which foods caused problems? (multiple answers allowed)	Egg / Milk / Wheat / Soy / Sesame / Crustaceans / Fish / Nuts / Peanuts / Buckwheat / Roe / Fruits / Other

**Food Allergy**	What type of food allergy was diagnosed by a doctor?	Immediate-type / Oral / FPIES / FDEIA / Unknown
**Food Allergy**	How was the food allergy diagnosed?	Symptoms + Blood test / Symptoms + Blood + Challenge / Blood only / Symptoms only / Avoided by choice
**Food Allergy**	Does your child carry an epinephrine auto-injector (EpiPen)?	Yes / No
**Food Allergy**	Has your child outgrown all food allergies?	Yes / No
**Conjunctivitis**	By age 6, has your child experienced persistent itchy eyes?	Yes / No
**Conjunctivitis**	In which months did the eye symptoms occur?	Jan–Dec (check all that apply)
**Conjunctivitis**	How much did eye symptoms interfere with your child's daily life by age 6?	Not at all / Slightly / Moderately / Severely
**Conjunctivitis**	Has your child ever been diagnosed with allergic conjunctivitis by a doctor?	Yes / No
**Birth & Family History**	What was your child's mode of delivery?	Vaginal / Cesarean
**Birth & Family History**	Does your child have a family history of allergy (father)?	Yes / No / Unknown + Type
**Birth & Family History**	Does your child have a family history of allergy (mother)?	Yes / No / Unknown + Type
**Birth & Family History**	Does your child have a family history of allergy (siblings)?	Yes / No / Unknown + Type

**Living Environment**	Is there a pet in your household?	Yes / No
**Living Environment**	Is there anyone in the household who smokes?	Yes / No
