# Supplementary material for: Comparison of Serum TARC Levels at Term‐Equivalent Age Between Preterm and Term Infants
Source: J Immunol Res. 2026 May 29;2026:3984014. doi: 10.1155/jimr/3984014 (PMC13239061; doi:10.1155/jimr/3984014)
Supplement: Supplementary file 2 — Supporting Information 2 Table S2: Main diagnoses in hospitalized term infants. [file JIMR-2026-3984014-s001.pdf]

Supplementary Table S2. Main diagnoses in hospitalized term infants.

Diagnosis	Number of cases
**Low birth weight**	131
**Neonatal respiratory disorders**	98
**Neonatal jaundice**	65
**Neonatal infection (including suspected cases)**	41
**Neonatal asphyxia**	40
**Neonatal hypoglycemia**	28
**Neonatal vomiting**	17
**Neonatal apnea**	13
**Poor weight gain**	8
**Neonatal melena**	8
**Neonatal heart disease**	6
**Neonatal convulsions**	5
**Feeding difficulty**	5
**Neonatal arrhythmia**	4
**Congenital hypothyroidism**	3
**Neonatal thrombocytopenia**	3
**Congenital renal disease**	2
**Neonatal cephalohematoma**	2
**Others: Myotonic dystrophy, Cardiac mass, Hypospadias, Ventriculomegaly, Birth from a fall, Proptosis, Hypothermia, Congenital dislocation of knee, suspected hemolytic anemia, Genital malformation, Abdominal distension, Intracranial cyst, Osteogenesis imperfecta, Drug withdrawal syndrome, Abdominal mass, Parietal bone fracture, Maternal herpes simplex infection,**	1 case each

**Meconium plug syndrome, Placental abruption, Intracranial hemorrhage, Dehydration**	
