# Supplementary material for: Comparison of Serum TARC Levels at Term‐Equivalent Age Between Preterm and Term Infants
Source: J Immunol Res. 2026 May 29;2026:3984014. doi: 10.1155/jimr/3984014 (PMC13239061; doi:10.1155/jimr/3984014)
Supplement: Supplementary file 3 — Supporting Information 3 Figure S3: Classification of term neonates into inflammatory and noninflammatory groups. Term neonates (n = 500) were categorized into inflammatory and noninflammatory groups based on their primary clinical diagnoses at the time of serum sampling. The inflammatory group (n = 169) included neonates with confirmed infection and clinical conditions potentially associated with systemic inflammatory responses, such as respiratory disorders (e.g., respiratory distress syndrome, transient tachypnea of the newborn, or meconium aspiration syndrome) and perinatal asphyxia. The noninflammatory group (n = 331) included neonates without these conditions. [file JIMR-2026-3984014-s002.pdf]

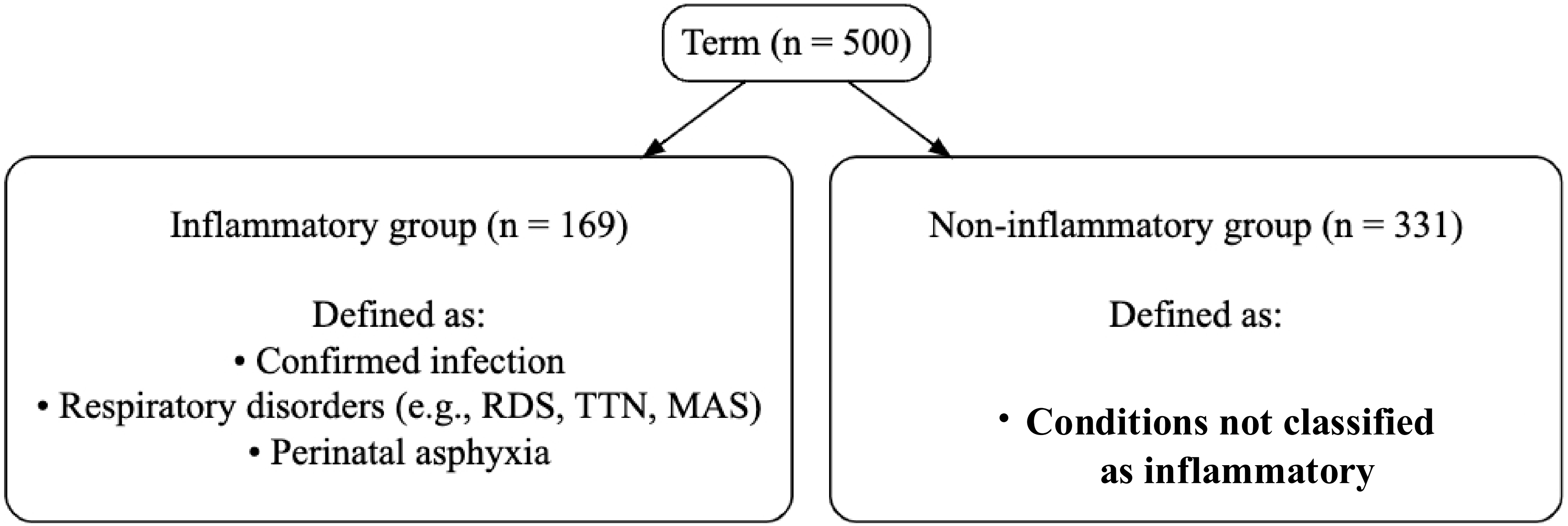

Supplementary Figure S3. Classification of term neonates into inflammatory and non-inflammatory groups.
Term neonates (n = 500) were categorized into inflammatory and non-inflammatory groups based on their primary clinical diagnoses at the time of serum sampling. The inflammatory group (n = 169) included neonates with confirmed infection and clinical conditions potentially associated with systemic inflammatory responses, such as respiratory disorders (e.g., respiratory distress syndrome, transient tachypnea of the newborn, or meconium aspiration syndrome) and perinatal asphyxia. The non-inflammatory group (n = 331) included neonates without these conditions.
