# Supplementary material for: Comparison of Serum TARC Levels at Term‐Equivalent Age Between Preterm and Term Infants
Source: J Immunol Res. 2026 May 29;2026:3984014. doi: 10.1155/jimr/3984014 (PMC13239061; doi:10.1155/jimr/3984014)
Supplement: Supplementary file 4 — Supporting Information 4 Figure S4: Serum TARC levels in inflammatory and non‐inflammatory groups among term neonates. Box plots show serum TARC levels (pg/mL) in term neonates classified into inflammatory (n = 169) and non‐inflammatory (n = 331) groups based on clinical diagnoses at the time of serum sampling. Differences between groups were assessed using the Wilcoxon rank‐sum test. [file JIMR-2026-3984014-s003.pdf]

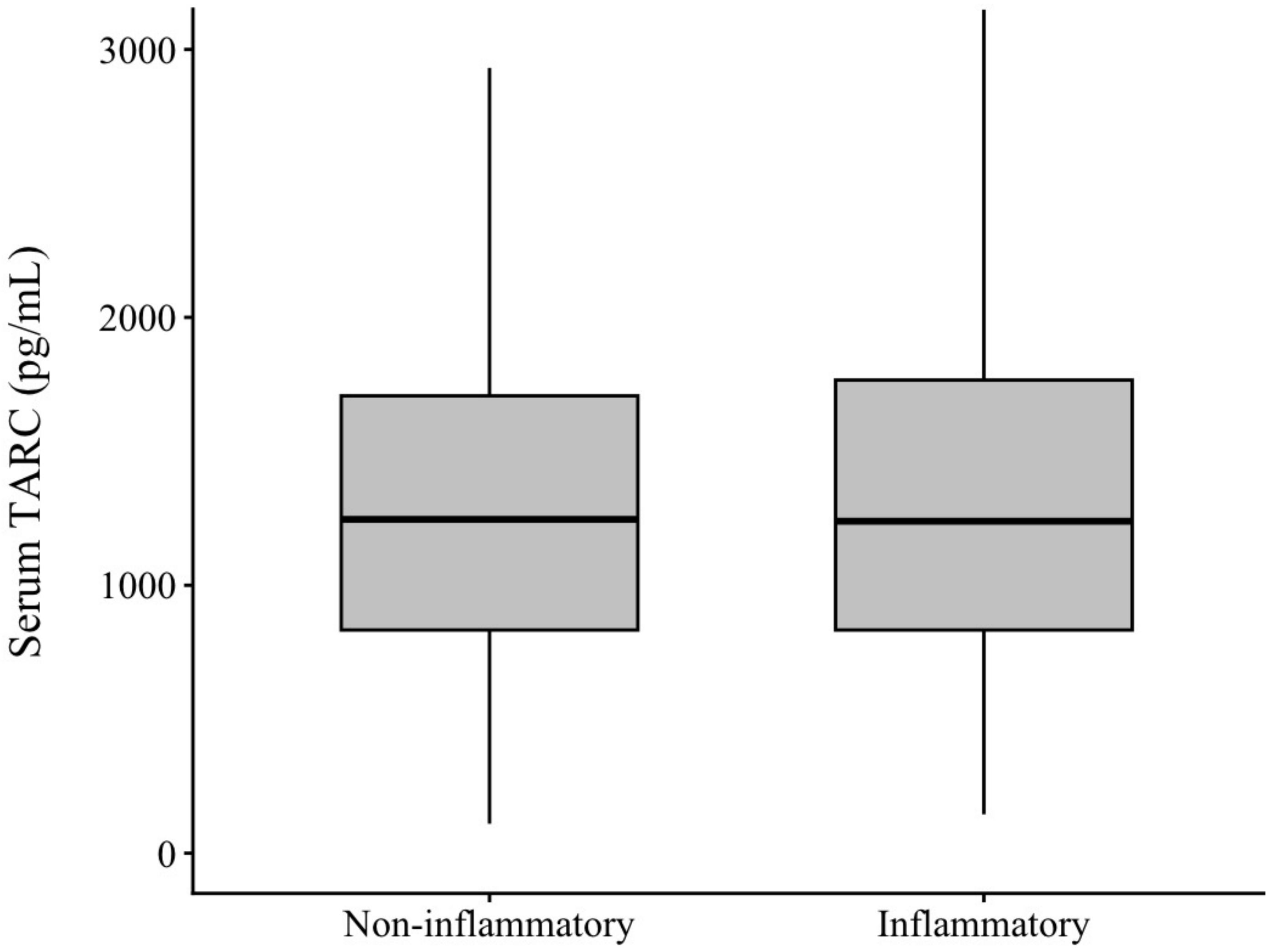

Supplementary Figure S4. Serum TARC levels in inflammatory and non-inflammatory groups among term neonates.
Box plots show serum TARC levels (pg/mL) in term neonates classified into inflammatory (n = 169) and non-inflammatory (n = 331) groups based on clinical diagnoses at the time of serum sampling. Differences between groups were assessed using the Wilcoxon rank-sum test.
