# Supplementary material for: Comparison of Serum TARC Levels at Term‐Equivalent Age Between Preterm and Term Infants
Source: J Immunol Res. 2026 May 29;2026:3984014. doi: 10.1155/jimr/3984014 (PMC13239061; doi:10.1155/jimr/3984014)
Supplement: Supplementary file 5 — Supporting Information 5 Table S3: Generalized linear model analysis of associations between serum TARC levels and clinical variables using a gamma distribution with a log link. [file JIMR-2026-3984014-s011.pdf]

Supplementary Table S3. Generalized linear model analysis of associations between serum TARC levels and clinical variables using a gamma distribution with a log link.

Variable	β estimate†	Standard error	*P* value
Birth weight (per 100g)	0.00052	0.00017	0.0016
Corrected Gestational age at sampling (Weeks)	-0.00824	0.00136	<0.001
Gestational age at birth (Weeks)	0.00623	0.00155	<0.001
Cesarean section	0.07888	0.03729	0.035
Sex (male)	-0.03845	0.0347	0.268
Small for gestational age	0.08415	0.05829	0.149
Systemic corticosteroid	0.05091	0.10271	0.620
Antibiotic use	0.0109	0.04591	0.812
Use of moisturizer	-0.00518	0.03577	0.885

† β estimate reflects the log-scale association with serum TARC levels.
