# Supplementary material for: Comparison of Serum TARC Levels at Term‐Equivalent Age Between Preterm and Term Infants
Source: J Immunol Res. 2026 May 29;2026:3984014. doi: 10.1155/jimr/3984014 (PMC13239061; doi:10.1155/jimr/3984014)
Supplement: Supplementary file 6 — Supporting Information 6 Table S4: Severity distribution of chorioamnionitis. [file JIMR-2026-3984014-s005.pdf]

Supplementary Table S4. Severity distribution of chorioamnionitis.

Stage (Blanc classification)	Extremely preterm (n=92)	Very preterm (n=137)	Total
None, n (%)	24 (26.1%)	40 (29.2%)	64 (27.9%)
1, n (%)	21 (22.8%)	35 (25.5%)	56 (24.5%)
2, n (%)	29 (31.5%)	38 (27.7%)	67 (29.3%)
3, n (%)	22 (23.9%)	24 (17.5%)	46 (20.1%)
