# Supplementary material for: Comparison of Serum TARC Levels at Term‐Equivalent Age Between Preterm and Term Infants
Source: J Immunol Res. 2026 May 29;2026:3984014. doi: 10.1155/jimr/3984014 (PMC13239061; doi:10.1155/jimr/3984014)
Supplement: Supplementary file 7 — Supporting Information 7 Figure S1: Serum TARC levels according to the severity of histological chorioamnionitis. Box plots show serum TARC levels (pg/mL) across four groups based on histological chorioamnionitis severity according to the Blanc classification: stage 0 (n = 64), stage 1 (n = 56), stage 2 (n = 67), and stage 3 (n = 46). Histological chorioamnionitis data were available for 233 infants in total. Differences among groups were not statistically significant (Kruskal–Wallis test, p = 0.467). The central line represents the median, box edges indicate the interquartile range (IQR), and whiskers represent 1.5 × IQR. [file JIMR-2026-3984014-s006.pdf]

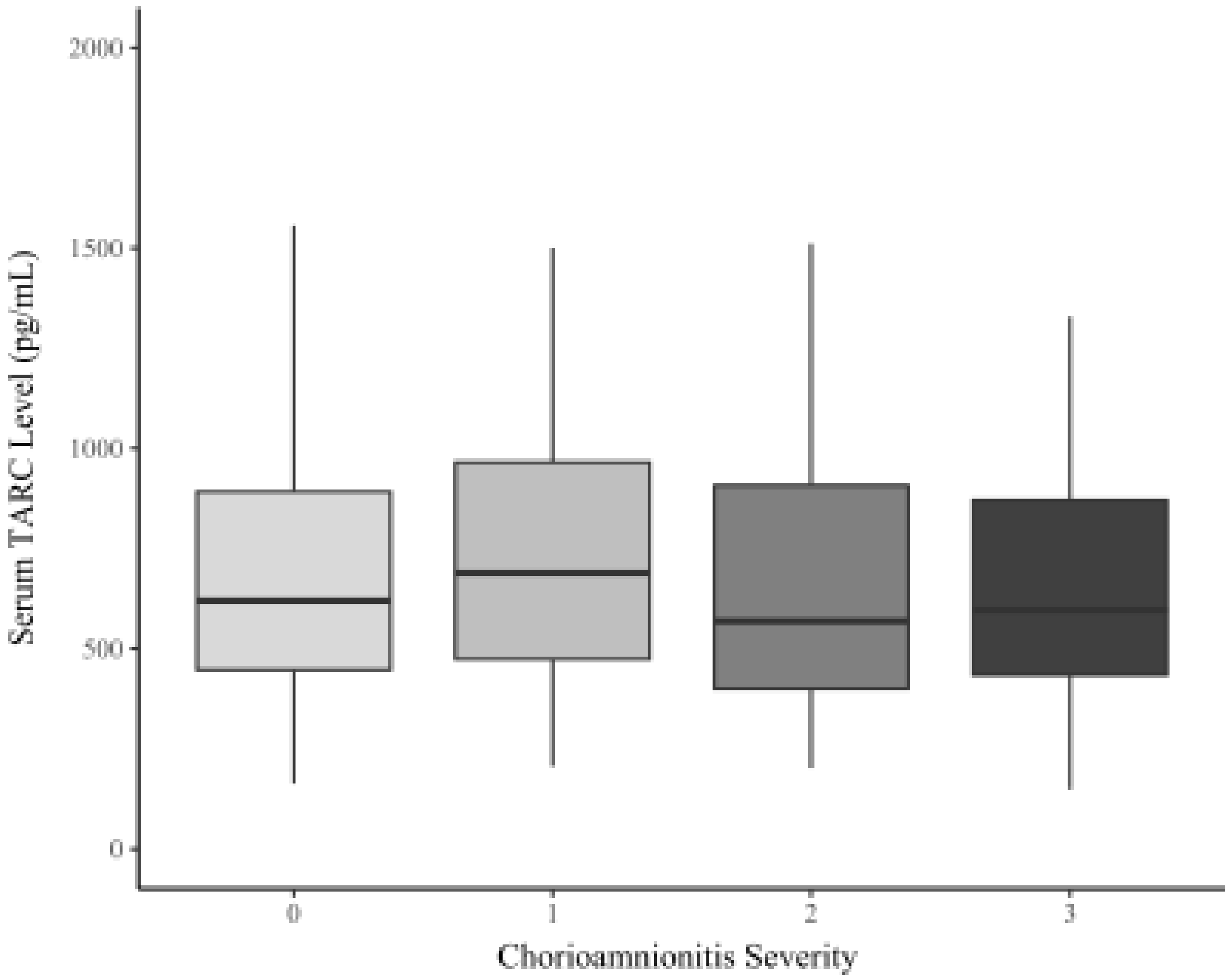

Supplementary Figure S1. Serum TARC levels according to the severity of histological chorioamnionitis
Box plots show serum TARC levels (pg/mL) across four groups based on histological chorioamnionitis severity according to the Blanc classification: stage 0 (n = 64), stage 1 (n = 56), stage 2 (n = 67), and stage 3 (n = 46). Histological chorioamnionitis data were available for 233 infants in total. Differences among groups were not statistically significant (Kruskal-Wallis test, $P = 0.467$). The central line represents the median, box edges indicate the interquartile range (IQR), and whiskers represent 1.5 × IQR.
