# Supplementary material for: Comparison of Serum TARC Levels at Term‐Equivalent Age Between Preterm and Term Infants
Source: J Immunol Res. 2026 May 29;2026:3984014. doi: 10.1155/jimr/3984014 (PMC13239061; doi:10.1155/jimr/3984014)
Supplement: Supplementary file 8 — Supporting Information 8 SFigure S2: Questionnaire follow‐up status by gestational age groups. At 6 years of age, follow‐up questionnaires were mailed to caregivers. The numbers of questionnaires returned, undelivered because of relocation, and nonresponses are shown for each gestational age group. Response rates were 49.3% in the extremely preterm group, 40.7% in the very preterm group, 37.4% in the moderate‐to‐late preterm group, and 28.4% in the term group. [file JIMR-2026-3984014-s007.pdf]

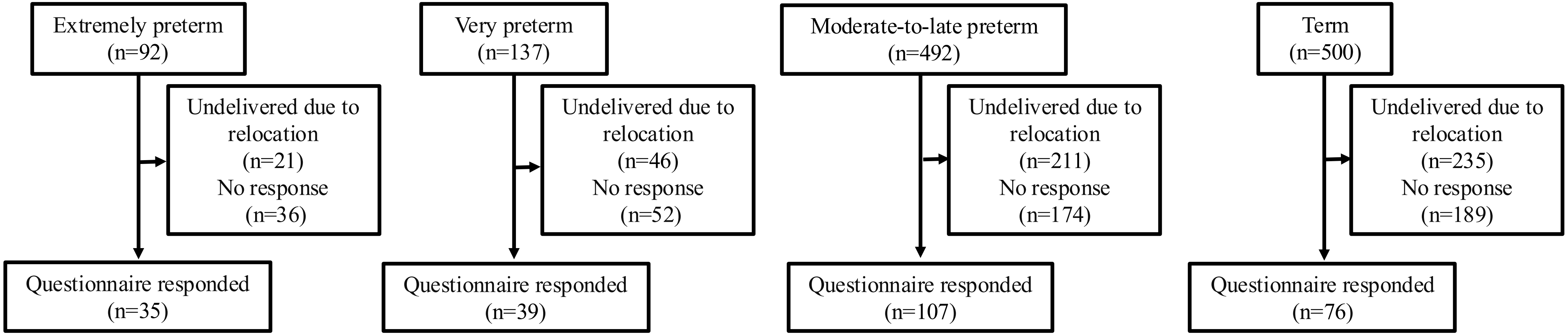

Supplementary Figure S2. Questionnaire follow-up status by gestational age groups.
At 6 years of age, follow-up questionnaires were mailed to caregivers. The numbers of questionnaires returned, undelivered because of relocation, and non-responses are shown for each gestational age group. Response rates were 49.3% in the extremely preterm group, 40.7% in the very preterm group, 37.4% in the moderate-to-late preterm group, and 28.4% in the term group.
