# Supplementary material for: Comparison of Serum TARC Levels at Term‐Equivalent Age Between Preterm and Term Infants
Source: J Immunol Res. 2026 May 29;2026:3984014. doi: 10.1155/jimr/3984014 (PMC13239061; doi:10.1155/jimr/3984014)
Supplement: Supplementary file 9 — Supporting Information 9 Table S5: Multivariable logistic regression analysis examining associations between parental allergic disease and atopic dermatitis. [file JIMR-2026-3984014-s008.pdf]

**Supplementary Table S5. Multivariable logistic regression analysis examining associations between parental allergic disease and atopic dermatitis.**

| Variable | Adjusted OR | 95% CI | *P* value |
|---|---|---|---|
| Preterm birth | 0.23 | 0.09–0.54 | <0.01 |
| Maternal allergic disease | 1.23 | 0.51–3.16 | 0.65 |
| Paternal allergic disease | 1.50 | 0.63–3.72 | 0.37 |
| Household smoking exposure | 0.74 | 0.26–1.88 | 0.54 |

Odds ratios (ORs) were estimated using multivariable logistic regression models adjusted for preterm birth, parental allergic disease, household smoking exposure, and household pet ownership. Estimates for household pet ownership were unstable because of sparse data and should be interpreted with caution.
