# Supplementary material for: Comparison of Serum TARC Levels at Term‐Equivalent Age Between Preterm and Term Infants
Source: J Immunol Res. 2026 May 29;2026:3984014. doi: 10.1155/jimr/3984014 (PMC13239061; doi:10.1155/jimr/3984014)
Supplement: Supplementary file 10 — Supporting Information 10 Table S6: Prevalence of allergic diseases and environmental factors at 6 years of age by gestational age groups. [file JIMR-2026-3984014-s009.pdf]

Supplementary Table S5. Prevalence of allergic diseases and environmental factors at 6 years of age by gestational age groups.

Variables	Extremely preterm (n=35)	Very preterm (n=37)	Moderate-to-late preterm (n=105)	Term (n=76)	*P* value†
Food Allergy, n (%)	1 (3%)	4 (10%)	10 (10%)	10 (13%)	0.27
Atopic Dermatitis, n (%)	3 (9%)	1 (3%)	7 (7%)	20 (27%)	<0.001
Allergic Rhinitis, n (%)	12 (34%)	16 (41%)	40 (38%)	36 (48%)	0.50
Bronchial Asthma, n (%)	15 (42%)	15 (38%)	24 (23%)	8 (11%)	<0.001
Allergic Conjunctivitis, n (%)	1 (3%)	10 (26%)	15 (14%)	10 (13%)	0.02
Maternal allergic disease, n (%)	20 (57%)	20 (51%)	59 (56%)	45 (65%)	0.32
Paternal allergic disease, n (%)	13 (37%)	19 (51%)	49 (47%)	28 (37%)	0.20
Home smoker, n (%)	7 (20%)	14 (36%)	24 (23%)	28 (37%)	0.20
Pet ownership, n (%)	8 (23%)	6 (15%)	16 (15%)	14 (19%)	0.71

†Fisher's exact test was used to compare categorical variables among gestational age groups.
