# Supplementary material for: Comparison of Serum TARC Levels at Term‐Equivalent Age Between Preterm and Term Infants
Source: J Immunol Res. 2026 May 29;2026:3984014. doi: 10.1155/jimr/3984014 (PMC13239061; doi:10.1155/jimr/3984014)
Supplement: Supplementary file 11 — Supporting Information 11 Table S7: Associations between serum TARC levels at term‐equivalent age and allergic outcomes at 6 years of age. [file JIMR-2026-3984014-s004.pdf]

Supplementary Table S6. Associations between serum TARC levels at term-equivalent age and allergic outcomes at 6 years of age.

Gestational age group	Bronchial asthma	Atopic dermatitis	Food allergy	Allergic conjunctivitis
Extremely preterm	NS	NS	NS	NS
Very preterm	Higher serum TARC levels in infants with bronchial asthma (*P* = 0.015)	NS	NS	NS
Moderate-to-late preterm	NS	NS	NS	NS
Term	NS	NS	NS	NS

Abbreviations: NS, not significant; TARC, thymus and activation-regulated chemokine.

P values were obtained using the Wilcoxon rank-sum test.
